# Structural and functional connectivity of the ascending arousal network for prediction of outcome in patients with acute disorders of consciousness

**DOI:** 10.1038/s41598-021-98506-7

**Published:** 2021-11-25

**Authors:** Cesar O. Enciso-Olivera, Edgar G. Ordóñez-Rubiano, Rosángela Casanova-Libreros, Diana Rivera, Carol J. Zarate-Ardila, Jorge Rudas, Cristian Pulido, Francisco Gómez, Darwin Martínez, Natalia Guerrero, Mayra A. Hurtado, Natalia Aguilera-Bustos, Clara P. Hernández-Torres, José Hernandez, Jorge H. Marín-Muñoz

**Affiliations:** 1 Department of Critical Care and Intensive Care Unit, Fundación Universitaria de Ciencias de La Salud (FUCS), Hospital Infantil Universitario de San José, Bogotá, Colombia; 2 Department of Neurological Surgery, Fundación Universitaria de Ciencias de La Salud (FUCS), Hospital de San José, Bogotá, Colombia; 3 Division of Clinical Research, Fundación Universitaria de Ciencias de La Salud (FUCS), Hospital de San José, Hospital Infantil Universitario de San José, Bogotá, Colombia; 4 Department of Biotechnology, Universidad Nacional de Colombia, Bogotá, Colombia; 5 Department of Mathematics, Universidad Nacional de Colombia, Bogotá, Colombia; 6 Department of Computer Science, Universidad Nacional de Colombia, Bogotá, Colombia; 7 Department of Computer Science, Universidad Central, Bogotá, Colombia; 8 Department of Radiology, Fundación Universitaria de Ciencias de La Salud (FUCS), Hospital Infantil Universitario de San José, Bogotá, Colombia; 9 Department of Psychology, Fundación Universitaria de Ciencias de La Salud (FUCS), Hospital Infantil Universitario de San José, Bogotá, Colombia; 10 Department of Neurology, Fundación Universitaria de Ciencias de La Salud (FUCS), Hospital Infantil Universitario de San José, Bogotá, Colombia; 11 Innovation and Research Division, Imaging Experts and Healthcare Services (ImexHS), Street 92 # 11-51, Of 202, Bogotá, Colombia

**Keywords:** Diffusion tensor imaging, Functional magnetic resonance imaging, Magnetic resonance imaging, Prognosis, Magnetic resonance imaging, Neurological disorders

## Abstract

To determine the role of early acquisition of blood oxygen level-dependent (BOLD) signals and diffusion tensor imaging (DTI) for analysis of the connectivity of the ascending arousal network (AAN) in predicting neurological outcomes after acute traumatic brain injury (TBI), cardiopulmonary arrest (CPA), or stroke. A prospective analysis of 50 comatose patients was performed during their ICU stay. Image processing was conducted to assess structural and functional connectivity of the AAN. Outcomes were evaluated after 3 and 6 months. Nineteen patients (38%) had stroke, 18 (36%) CPA, and 13 (26%) TBI. Twenty-three patients were comatose (44%), 11 were in a minimally conscious state (20%), and 16 had unresponsive wakefulness syndrome (32%). Univariate analysis demonstrated that measurements of diffusivity, functional connectivity, and numbers of fibers in the gray matter, white matter, whole brain, midbrain reticular formation, and pontis oralis nucleus may serve as predictive biomarkers of outcome depending on the diagnosis. Multivariate analysis demonstrated a correlation of the predicted value and the real outcome for each separate diagnosis and for all the etiologies together. Findings suggest that the above imaging biomarkers may have a predictive role for the outcome of comatose patients after acute TBI, CPA, or stroke.

## Introduction

Predicting neurological outcomes and mortality in patients with acute disorders of consciousness (DOCs) is challenging^1–3^, and clinicians continue to question complex connection impairments of the ascending arousal network (AAN) in comatose patients^2,4^. Unfortunately, current clinical and radiological tools are not reliable for detecting consciousness or predicting recovery in those with either severe traumatic brain injury (TBI)^5^, cardiac arrest^3,6,7^, or stroke^7,8^. To this end, multiple clinical and radiological tests have been proposed for assessing patients with DOCs, including bedside behavioral assessment^9^, electroencephalography (EEG)^10^, task-based functional magnetic resonance imaging (fMRI)^11^, resting-state-fMRI (rsfMRI)^6,7,12^, diffusion weighted imaging (DWI)^13^, diffusion tensor imaging (DTI)^1,2,7,8,14^, and different combinations of these approaches^5,12,15^. In the absence of reliable prognostic tests, the clinician’s judgment, experience, and communication skills may influence a family’s decision about life-sustaining therapy and lead to premature care decisions before a patient’s prognosis becomes clear^15^. In this regard, investigations have focused on diagnostic tests that might objectify this initial prediction assessment of consciousness outcome^2,4,6,7^.

The AAN is an essential component of human consciousness and is formed by a group of subcortical pathways connecting the rostral brainstem tegmentum to the hypothalamus, thalamus, and basal forebrain^2,14^. It has been described that DOCs after TBI, cardiac arrest, or stroke are related to axonal injury within the AAN^1,14,16^. Additionally, clinical and electrophysiological evaluations are insufficient and might be biased by sedation or any clinical condition, such as aphasia^17^. Accordingly, there is uncertainty concerning long-term effects in a broad spectrum of cognitive, behavioral, and functional impairments^17^. Overall, more specialized tests derived from MRI may be able to better characterize microstructural disturbances.

Blood oxygen level-dependent (BOLD) imaging utilizes a gradient-echo imaging sequence with parameters sensitive to the oxygen state of hemoglobin, which is used as contrast to delineate regional brain activity^18^. BOLD imaging allows for the analysis of both task-based fMRI and rsfMRI. rsfMRI itself can be used for studying different resting-state neural networks (RSNs) to establish functional connectivity in patients with a DOC^12^. On the other hand, DTI uses anisotropic diffusion to estimate the organization of brain tissue. Additionally, structural analysis of white matter (WM) with DTI techniques, including diffusion tensor tractography (DTT), has allowed physicians to scrutinize the anatomy of the AAN^1,8,14,16,19^, revealing the structural connectivity of this network^1,20^. Both tools can be employed to determine ascending and descending structural and functional connectivity between AAN brainstem nuclei and many different cortical areas^12^. Nevertheless, the exact biological nature of the structural and functional injury that leads to a DOC remains uncertain. Multiple efforts to elucidate the origin of impaired consciousness have led to the proposition of a compromised state for multiple cortical and subcortical areas and networks that may be involved in this process, including the brainstem^21^, thalamus^6,22^, hypothalamus^1^, frontal basal cortex^1,4,5,14^, and other association areas in the parietal lobe^6^. However, varying impairment in these areas may induce any DOC regardless of the etiology of the injury. In this regard, the aim of the present study was to analyze a combination of structural and functional information of the AAN obtained from both DTI and BOLD acquisitions to determine whether early acquisition of DTI and BOLD techniques for analysis of structural and functional connectivity of the AAN can predict neurological outcomes in terms of consciousness in patients with DOCs after TBI, cardiopulmonary arrest (CPA), or stroke.

## Results

### Clinical features

**Figure 1:**
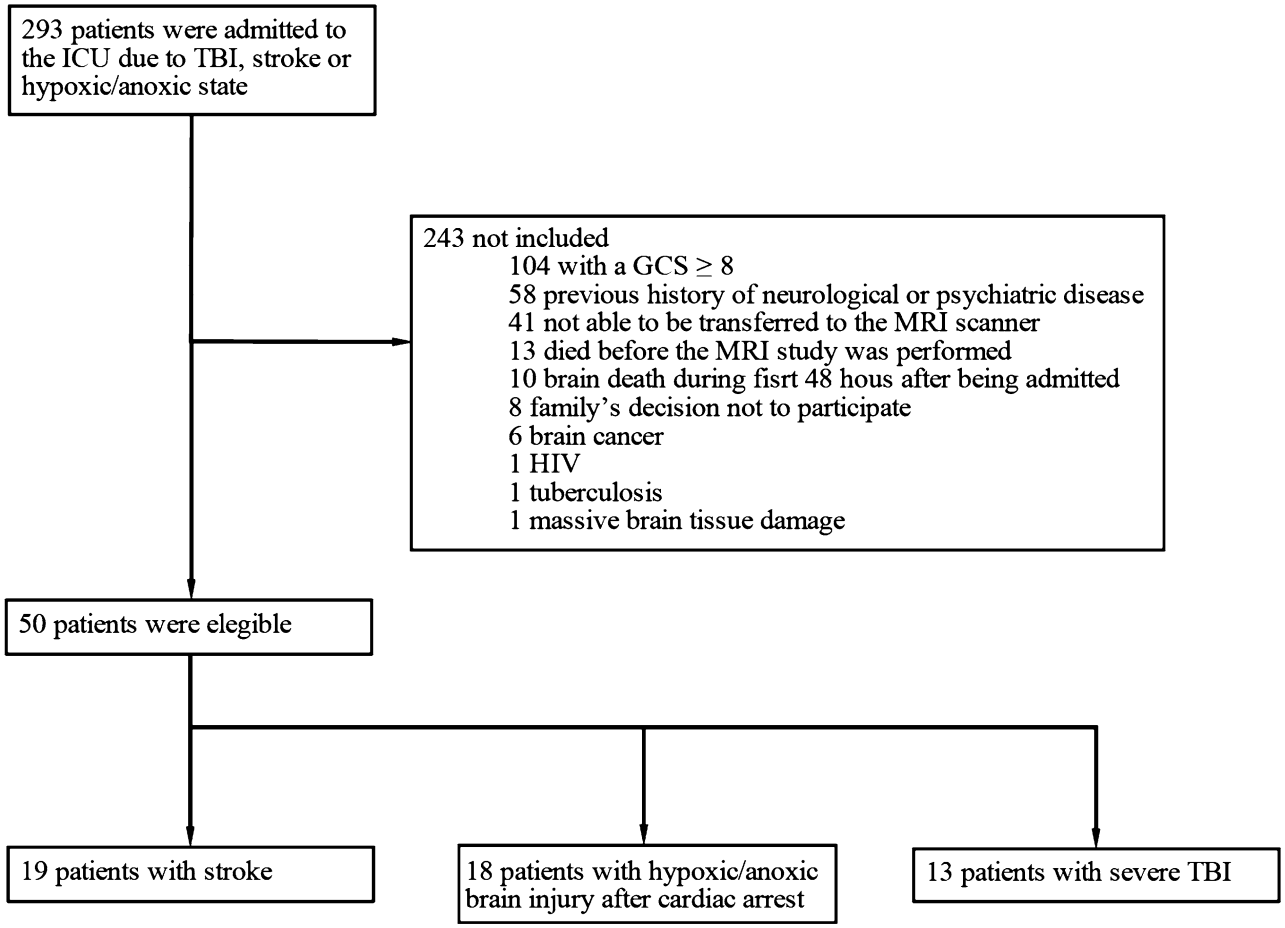
Description of the enrollment of patients in the study.

Between October 2017 and January 2020, a total of 293 patients were assessed for eligibility criteria, of whom 50 were enrolled. Of the 243 excluded subjects, 104 were excluded for having a GCS score 8 or higher, 58 due to a previous history of any neurological or psychiatric disease, 41 because they were not able to be transferred to the MRI scanner due to their medical condition, 13 because MRI was not performed before death, 10 due to radiologically confirmed brain death during the first 48 h after being admitted to the ICU, eight due to the family’s decision not to participate, and nine due to other reasons (Fig. 1). The median age of the enrolled patients was 64 years (IQR 49–74), and 27 (54%) were female. Nineteen patients (38%) were admitted with stroke, 18 (36%) with hypoxic-ischemic brain injury after cardiac arrest, and 13 (26%) with severe TBI. In regard to the state of consciousness, 23 patients were in coma (46%), 11 in MCS (22%), and 16 in UWS (32%). The median length of stay in the ICU was 13.2 days (IQR 5.1–21.3). The overall median ICU admission GCS score was 6 (IQR 3–8); the UWS group had a median score of 8 (IQR 4–8), the coma group a median score of 6 (IQR 3–7), and MCS a median score of 5 (IQR 4–8). The 27 patients (54%) who were discharged from the ICU had a median GCS score of 9 (IQR 3–12) (Table 1). Among them, 12 died during the follow-up before the neuropsychological evaluation in an outpatient setting. Additionally, 5 patients were lost from the study due to loss of contact with their surrogates (4 from the coma group and 1 from the UWS group).

**Table 1:** Clinical and demographic features according to state of consciousness after ICU admission.

	Coma (n = 23)	MCS (n = 11)	UWS (n = 16)	Total (n = 50)	p value
Age *median* (IQR)	62 (44–76)	65 (57–69)	66 (47–74)	64 (49–74)	0.62
**Sex**					0.06
Female (%)	12 (52,2)	6 (54.5)	9 (56.2)	27 (54)	
Male (%)	11 (47.8)	5 (45.5)	7 (43.8)	23 (46)	
**Education**					0.98
Illiterate (%)	1 (4.3)	0 (0)	0 (0)	0 (0)	
Primary school (%)	7 (30.4)	3 (27.3)	7 (43.8)	17 (34)	
High school (%)	8 (34.8)	3 (27.3)	5 (31.2)	16 (32)	
Technical (%)	2 (8.7)	1 (9.1)	1 (6.25)	4 (8)	
Graduate school (%)	5 (21.7)	4 (36.4)	3 (37.5)	12 (24)	
**Comorbidities**					0.82
Hypertension (%)	7 (30.4)	7 (63.6)	8 (50)	22 (44)	
Diabetes mellitus (%)	6 (26.1)	3 (27.3)	3 (18.8)	12 (24)	
Hypothyroidism (%)	2 (8.7)	2 (18.2)	3 (18.8)	7 (14)	
Dyslipidemia (%)	1 (4.3)	1 (9.1)	0 (0)	2 (4)	
Acute myocardial infarction (%)	0 (0)	1 (9.1)	1 (6.3)	2 (4)	
Other (%)	5 (21.7)	5 (45.5)	11 (68.8)	21 (42)	
ICU admission GCS score *median* (IQR)	6 (3–7)	5 (4–8)	8 (4–8)	6 (3–8)	0.08
**Etiology of the DOC**					
Stroke	7 (30.4)	5 (45.5)	7 (43.8)	19 (38)	0.59
Trauma	6 (26.1)	4 (36.4)	3 (18.8)	13 (26)	
Cardiopulmonary arrest	10 (43.5)	2 (18.2)	6 (37.5)	18 (36)	
**Intracranial pressure**					
ICP monitoring (%)	2 (8.7)	1 (9.1)	1 (6.25)	4 (8)	0.95
Intracranial hypertension (%)	2 (8.7)	2 (18.2)	1 (6.25)	5 (10)	0.57
Mechanical ventilation (%)	22 (95.7)	10 (90.9)	14 (87.5)	46 (92)	0.65
Sepsis (%)	8 (34.8)	3 (27.3)	8 (50)	19 (38)	0.45
**Vital status**					
Alive (%)	13 (56.5)	9 (81.8)	5 (31.3)	27 (54)	0.03
Deceased (%)	6 (26.1)	2 (18.8)	10 (62.5)	18 (36)	
*Transferred to a different institution (%)	0 (0)	1 (9.1)	2 (12.5)	3 (6)	
GCS score at discharge *median* (IQR)	6 (3–11)	3 (3–14)	11 (9–12)	9 (3–12)	0.15

*Expenses not covered by their health insurance company in our institution.

P values correspond to comparative measures among the three patient groups.

### Structural and functional connectivity

#### Univariate analysis

**Figure 2:**
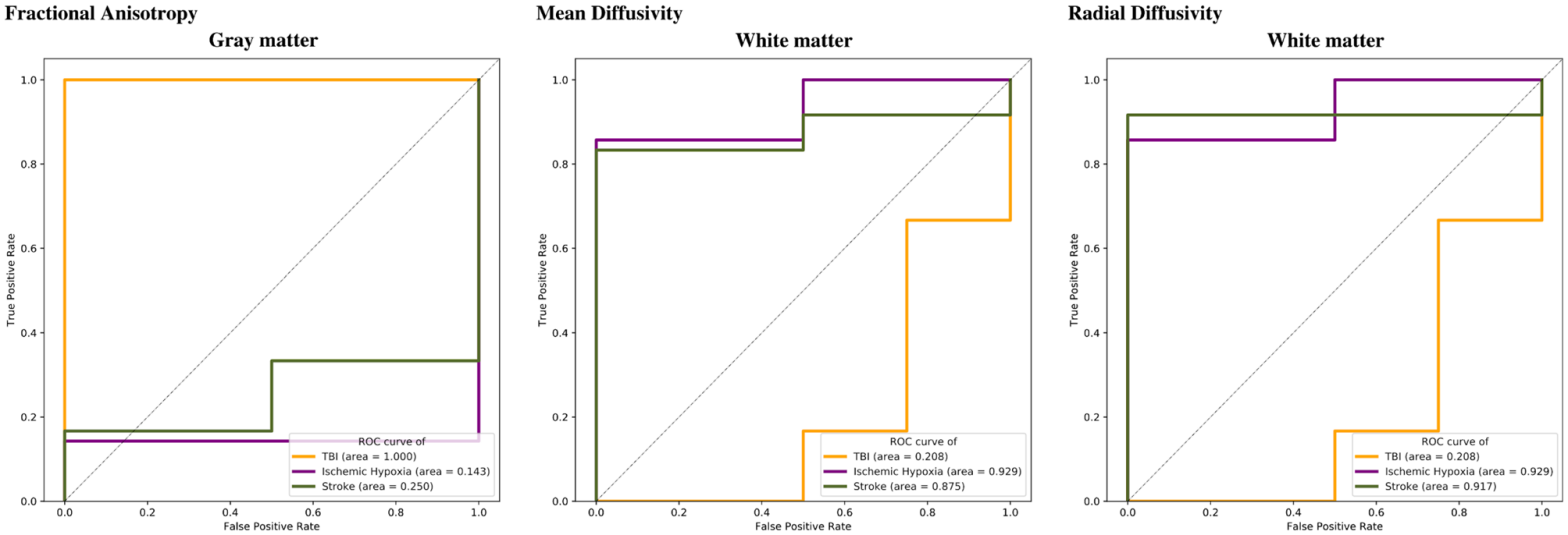
Receiver operating characteristic (ROC) curves of single measurements for different cortical and subcortical areas. ROC curves demonstrate the possible predictive value of data comparing true positive and false positive rates for fractional anisotropy, mean diffusivity, and radial diffusivity among patients with TBI (yellow), cardiac arrest (purple), and stroke (green).

A set of 60 individual imaging measurements was completed. However, only sixteen had an area under the curve (AUC) $\ge \hspace{0.17em}$0.80. Thus, these specific features were explored as possible predictors with at least an accuracy of 80%. A remarkable finding is that some single regions might serve as biomarkers of consciousness at ICU discharge. Figure 2 illustrates the potential use of single imaging measurements in specific regions to predict the patient’s consciousness at ICU discharge. The ROC curve indicates possible use as an isolated approach for measuring specific regions to predict GCS score at ICU discharge.

**Figure 3:**
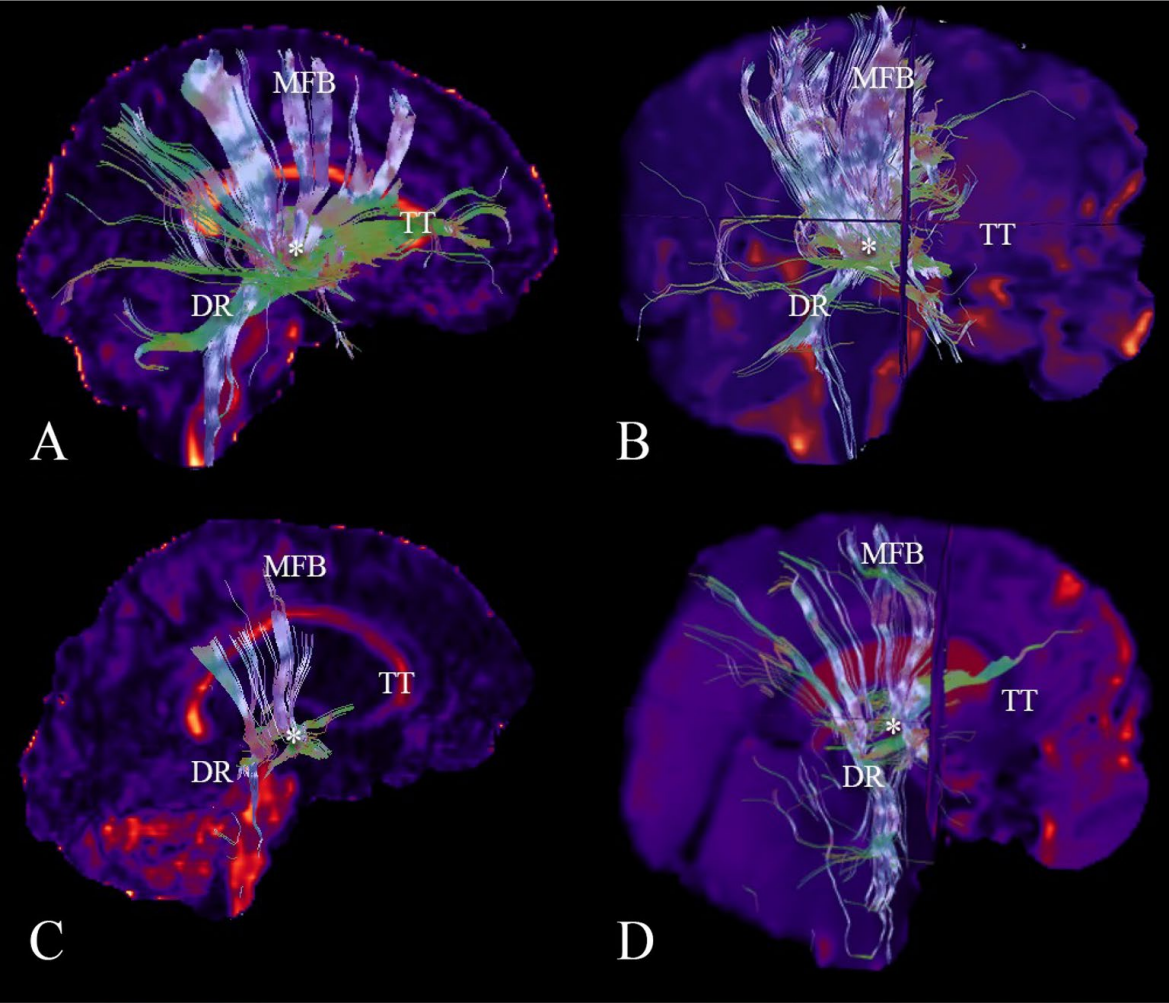
Reconstruction of the tractography of ascending arousal network. (**A**) A complete reconstruction of the AAN of a normal subject is demonstrated. Major components including the medial forebrain bundle (MFB) projecting to the dorsal frontal cortex, the dorsal raphe (DR), the thalamic-hypothalamic complex (asterisk), and superior and inferior tegmental tracts (TT) projecting to the basal frontal cortex are shown. (**B**) A reconstruction of the AAN of a comatose patient after a severe traumatic brain injury is observed, denoting a destruction of the tegmental tracts. (**C**) A reconstruction of the AAN of a patient after a stroke demonstrates a disruption of the tracts in the length of the MFB, the fibers of the DR, and the tegmental tracts. (**D**) A reconstruction of the AAN is shown in a comatose patient after a cardiac arrest, demonstrating a decrease in the number of fibers of all components of the AAN.

Based on separate analysis for each variable, the gray matter (GM) FA was found to be a possible predictor in the setting of TBI. The AD, MD, and RD in both the GM and WM, as well as functional connectivity in the PON, and the number of fibers in the locus coeruleus (LC) and the parabrachial complex (PC) are possible predictors in the setting of CPA. Measurements of MD in the whole brain, WM, GM, MRF and PON, of AD in the WM, GM, and MRF, and the RD in the whole brain, WM, and MRF were found to be possible predictors of the state of consciousness at ICU discharge in the setting of a stroke. Separate analysis of automatic DTI, tractrography (Fig. 3) and BOLD measurements showed no predictive value for the state of consciousness at ICU discharge.

#### Structural and functional connectivity—multivariate analysis

**Figure 4:**
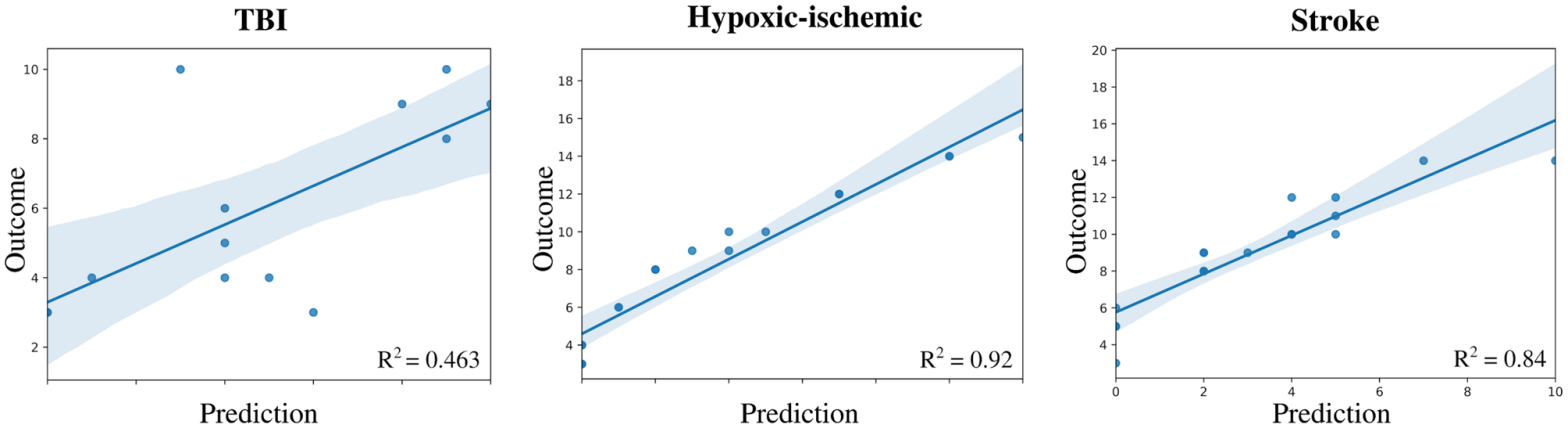
Multivariate analysis for prediction of outcomes of consciousness by etiology. In the three scenarios (traumatic brain injury, cardiac arrest, and stroke), the outcome for each patient is demonstrated by a linear model. The curves show a direct correlation between imaging and prediction of consciousness outcome, with corresponding R^2^ values of 0.463, 0.92 and 0,84, respectively.

**Figure 5:**
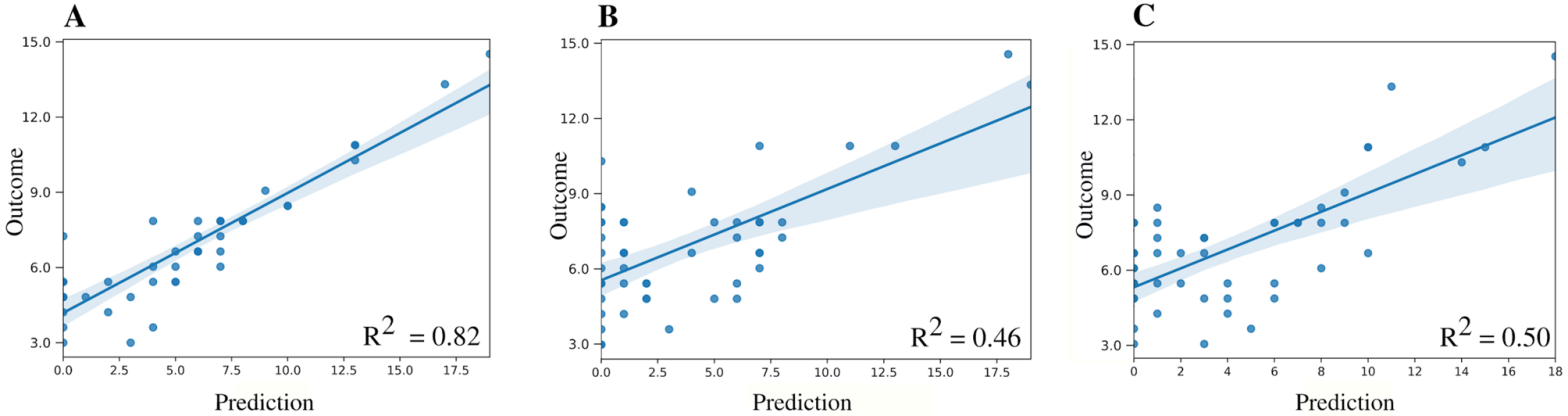
Multivariate prediction at the group level. The relationship between outcome and prediction of the general linear model is demonstrated using (**A**) both the structural and functional descriptors together, (**B**) the functional descriptors alone, and (**C**) the structural descriptors alone. The corresponding R^2^ values are 0.82, 0.46 and 0.5, respectively.

Figure 4 illustrates the results obtained by GLM for each separate diagnosis; independent variables included the confounding variables and those features obtained from DTI and BOLD acquisitions. This figure shows the correlation between the real outcome and the value predicted by the model. In the setting of TBI, the model reached an adjustment defined by the metric R2 = 0.463; it was 0.84 for stroke and 0.92 for CPA. Moreover, Fig. 5 denotes the correlation between the outcome and the model’s predicted value from a global aspect, grouping patients with TBI, CPA, and stroke into one group. Three different scenarios were explored: (1) including both structural (DTI) and functional (BOLD) features, (2) functional features alone, and (3) structural features alone. The R2 values obtained for each scenario were 0.82, 0.463 and 0.5, respectively. These trends show the great importance of combining both features.

### Neurological outcomes

A total of 30 of the 50 patients died during follow-up before the neuropsychological evaluation. Of those remaining, five were lost during follow-up. Tests were performed for the remaining 15 patients. The NeuroPsi demonstrated that 9 (60%) of the patients had normal psychological behavior, 1 (6.6%) presented moderate psychological sequelae, and 5 (33.3%) presented severe cognitive sequelae. Additionally, 40% presented abnormal orientation, 3 (20%) had severe compromise of their attention/concentration, and 4 (26.7%) and 5 (33.3%) had moderate and severe visual memory impairment, respectively. On the other hand, the MoCa test showed that 11 (73.3%) patients had cognitive deficits; 4 (26.7%) had normal scores. Visual memory showed higher cognitive compromise, while the best performance was observed for language, attention, and orientation.

## Discussion

In the last decade, multiple efforts have been made to characterize AAN, aiming to provide information to predict neurological outcomes in patients with severe brain injury and consequent DOCs^8,14,16,19^. The advancement of MRI techniques has allowed analysis of the anatomic and functional connectivity of the human brain^4,12,23,24^. In combination with the information acquired from DTI and BOLD, analysis of the AAN has revealed bidirectional connectivity between AAN nuclei in the brainstem and diencephalon and other multiple areas over the brain cortex^12^. Edlow and Fins^15^ proposed the existence of covert consciousness, which has been defined by the presence of command-following on task-based fMRI or EEG in patients whose bedside behavioral diagnosis suggests different DOCs, such as coma, vegetative state (VS), or a low-level MCS. Many questions arise regarding whether these patients may preserve consciousness, whether this covert consciousness might evolve and whether patients may recover full consciousness in short-, middle-, or long-term follow-up.

A recent study demonstrated that personalized connectome mapping may help to guide physicians to promote the recovery of patients with DOCs^4^. Our study enhances the importance of some specific values in regions that may be predictive of outcomes in comatose patients and adds remarkable information to guide personalized functional connectivity mapping. We would like to emphasize that despite the separate analysis of DTI information, the reconstruction of the tractography of AAN pathways and rsfMRI analysis did not show statistically significant values as predictors alone, but the combination of these measures can help with regard to correlation of the clinical, anatomical, and functional status of the AAN (R2 = 0.82 value in the plot A compared to plot B and C in Fig. 4). Our study contributes to the demonstration of specific targets that make the processing of valuable information from advanced MRI techniques easier and shorter.

DTI involves several metrics to quantify the degree and direction of water diffusion, as follows^25^: the FA, which measures directional coherence of water diffusion and reflects the degree of structural integrity and myelination of WM^26^; the MD, which represents the rate of net diffusion of molecules^27,28^; and the AD and RD, which also describe the diffusion direction and magnitude^28,29^. Our findings suggest that analysis of functional connectivity of the AAN at specific features of the DTI may serve as a biomarker to predict the state of consciousness after ICU stay depending on the etiology of the injury: the AD, MD, and RD of the GM and WM may be predictive in the setting of CPA; the FA of the GM in TBI; and the AD, MD, and RD of the whole brain, GM and WM in CPA. Such analysis of the AAN with DTI seems to fill the gap in predicting outcomes in comatose patients by assessing WM damage.

Patients with TBI present microstructural damage produced by diffuse axonal injury, which is caused by shear-strain deformation that develops upon exposure to rotational acceleration forces that damage the axon at the node of Ranvier^30^. These changes can be assessed with DTI metrics. Some authors have reported that FA values in WM are decreased in patients with TBI and impaired consciousness^1,31–33^. Our results suggest that FA values of the GM should also be evaluated for purposes of outcome prediction. We found this information relevant, as it should be expected that FA values are likely found impaired in the WM due to DAI. As DTI is not exact, it would be also considered that this must be influenced by DAI within the cortico-subcortical junction. In addition, it has been recently suggested that FA values can also be used in the GM for the quantification of the compression of a tissue, this is especially important for patients with severe structural damage^34^. Furthermore, a recent meta-analysis showed that the degree of correlation between FA values and consciousness level varies among brain regions^29^. This finding may be related to the integration of information coming from DTI and rsfMRI, which adds fundamental information for a complete analysis. For future research purposes, further investigations should examine FA values in different cortical and subcortical areas, such as the corpus callosum and the internal capsule, which are areas where FA values appear to have a good correlation with the level of consciousness in patients with TBI^29^.

On the other hand, during CPA, cessation of cerebral oxygen delivery occurs with resulting neuron ischemia and cell death within minutes^35^. In animal studies, a statistically significant decrease in WM anisotropy was observed at 43 h following transient acute cerebral hypoxia–ischemia, whereas the degree of GM anisotropy did not change significantly^36^. DTI metrics in the WM and GM showed a significant predictive value in our study, as did some pontine areas such as the PON, the locus coeruleus, and the parabrachial complex. These areas comprehend specific excitatory functions. The outputs of the locus coeruleus form an important contribution to the generalized neural activation underlying the maintenance of arousal^37^, whereas the parabrachial complex acts as a sensory relay of interceptive and exteroceptive inputs relevant for multiple aspects of autonomic control, such as respiration and thermoregulation^38^. Measurements of functional connectivity of the PON and the number of fibers of the locus coeruleus and the parabrachial complex were shown to predict outcome in our study. Nevertheless, further studies are needed to compare both consciousness and other autonomic functions in patients with compromised nuclei after CPA.

In ischemic stroke, cerebral ischemia arises from a reduction in the delivery of oxygen and nutrients to brain tissue due to obstructed blood flow, which results in cellular swelling and a decrease in the extracellular volume fraction^39^. Ischemia affects tissue water diffusion via the presence and orientation of barriers to translational motion, such as cell membranes and myelin fibers^40^; in a hemorrhagic stroke, Wallerian degeneration of the GM and WM in intimate relation to the hematoma occurs. Multiple mechanisms mediate injury in hemorrhagic stroke, including mechanical mass effects, inflammation, cellular toxicity, and edema around the periphery of the hematoma^41^. DTI metrics show that decreases in the ADC coincide with the onset of acute brain edema in ischemic stroke^42,43^. During the acute (24 h) and subacute (3–5 days) phases, WM ADC decreases more than does GM ADC^40^. Our findings indicate that the whole brain should be analyzed, including the WM and GM, for an adequate investigation of AAN patency. Contrary to expectations, damage may only be present in the site of the lesion and its peripheral structures, but the prediction of recovery may include the capacity of recovery of other areas within the brain, such as the anterior forebrain mesocircuit and the frontoparietal network, which are interconnected areas that increase their activity and functional connectivity when patients transition from coma to UWS or MCS and finally to full consciousness^44,45^. Despite stroke patients rarely progress to a PDOC, we found that for ischemic and hemorrhagic stroke patients, the MD, AD, and RD were fundamental for outcome prediction when analyzing the whole brain, GM, and WM. This implies that different cortical areas and the connections between them and connections with brainstem structures should be included for prediction analysis. Only specific brainstem areas such as the MRF and the PON demonstrated a remarkable role in prediction. The PON and MRF are part of the AAN, and dysfunction of their connections with some thalamic and hypothalamic areas has been reported to play a role in the origin of DOCs^46^.

Although neural injury differs following CPA, TBI, ischemic stroke, and hemorrhagic stroke^47^, this study demonstrates the predictive value of different biomarkers using both DTI and BOLD for neurological outcomes in comatose patients independent of etiology. This could be explained by the fact that there appears to be a common pathophysiological mechanism underlying DOCs: the withdrawal of excitatory synaptic activity across the brain produced by deafferentation (disconnection of afferent inputs) or disfacilitation (downregulation of neuronal firing rates) of neocortical, thalamic and striatal neurons^46^. Our multivariate analysis for each independent etiology as well as all patients together exhibited good correlation between the predictive value of the tests and the real outcome of the patients. It is also important to note that some cerebrum areas seem to be recurrent as possible biomarkers for outcome, including the GM, WM, MRF, and PON. Regardless, these separate measurements cannot be considered as a group that may affect the final prediction calculation. Although many questions remain regarding which patients with DOCs have the potential for recovery or may die, this study highlights the potential utility of AAN connectivity analysis with DTI and BOLD, which revealed possible biomarkers in comatose patients by identifying the most vulnerable areas of damage that may predict recovery of consciousness or death. However, it is important to mention that despite the classification of patients into different DOC groups was performed before completing a 4-week period where a diagnosis is more stable, a complementary information was used in terms of neurological status for prediction purposes including GCS score at ICU discharge to improve the analysis of the results. Thus, this classification process may bias the prediction potential of these biomarkers and the general analysis of the data. More studies are needed to find cutting-edge targeted treatments with the potential to reactivate injured neural networks and promote recovery of consciousness.

## Conclusions

Our findings suggest that early acquisition of BOLD and DTI for evaluation of structural and functional connectivity of the AAN may represent a tool for predicting outcome in patients with impaired consciousness after acute TBI, CPA, or stroke. DTI and BOLD analysis represent an observer- and operator-dependent task despite the automatic data processing involved, and clinical decision making must be made by physicians in a case-by-case manner.

### Limitations

The limited number of patients recruited for this study represents a notable limitation. Although different etiologies were included, a separate analysis of each group was performed. This study sought to elucidate the prognostic value of early DTI and BOLD acquisitions, yet there are multifactorial limitations to enrolling comatose patients, including the high mortality in these scenarios as well as the social and economic background of a middle-income country. In addition, as this study lacks EEG data, further studies are needed to compare EEG findings with radiological biomarkers. This study lacks from standard scales evaluation of prolonged DOC at the discharge and follow up, making the analysis limited in terms of specific neurological status, but it indexes the GSC score allowing a more general outcome prediction. In addition, it’s important to mention that the acquisition of images as well as the time for ICU discharge could be close in timeline and this could influence on the outcome assessment and results could be translated at some point into a diagnostic approach as well. Finally, specific analysis of the emotional, behavioral, and general neurological outcomes should also be addressed for targeted therapeutic assessment.

## Methods

### Clinical data and study design

This is a prospective, observational, cohort-type diagnostic test study. Patients admitted to the ICU with acute DOC after CPA, stroke or TBI who stayed in the ICU for more than 48 h were enrolled. Ten healthy volunteer adult subjects were recruited and analyzed as the control group for neuroimaging preprocessing calibration, including the automatic processing for delineation of the regions of interest (ROIs)^12^. Patients were admitted to the Hospital Infantil Universitario de San José ICU between October 2017 and January 2020. Inclusion criteria included patients over 18 years old, with either CPA treated within our institution with successful cardiopulmonary resuscitation, stroke (ischemic or hemorrhagic), or TBI, with a neurological evaluation prior to ICU admission consisting of coma (defined as Glasgow Coma Scale [GCS] score of ≤ 8/15 without eye opening) after the initial resuscitation and who could be transferred to the MRI scanner [median of 9 days (IQR 6–16) for acquisition after the event]. Exclusion criteria included patients diagnosed with brain death within the first 48 h of admission to the ICU, those with severe TBI who were considered to be “nonsalvageable” by the neurosurgery staff, and those who had any medical history of a neurological entity prior to the event (e.g., trauma, degenerative disease), and those whose family decided to withdraw them from the study at any time during the follow-up period. Written informed consent for inclusion in the study was obtained from a surrogate for each patient. Authorization by our Institutional Ethics Board to include information for the subjects was requested. This research was performed in accordance with the Declaration of Helsinki. This prospective study was approved by our Institutional Review Board (*Comité de Ética en Investigación con Seres Humanos—CEISH*).

### Neurological outcomes

The initial bedside cognitive and behavioral assessment was performed by a neurologist (J.H.) in the first 24 h or up to 3 days after the event whenever possible. The following tests were added for assessment by the patient’s family: Lawton-Brody Instrumental Activities Scale, Frontal Systems Behavior Scale, and Memory Scale. Any sensory perception disorder was ruled out, and the family was asked about the patient's previous cognitive condition in relation to symptoms associated with previous behavioral changes, schooling, and occupation or any conditions that may bias the cognitive assessment. A subsequent evaluation was made by a neurologist (J.H.) and the ICU staff within the first 7–10 days using the Coma Recovery Scale- Revised (CRS-R) and Full Outline of Unresponsiveness (FOUR) after the initial injury or at the time of discharge from the ICU in the case of a short length of ICU stay. After the second assessment, the patients were categorized into those with coma, unresponsive wakefulness syndrome (UWS) (defined as a state of wakefulness without awareness in which there is preserved capacity for spontaneous or stimulus-induced arousal, evidenced by sleep–wake cycles and a range of reflexive and spontaneous behaviors, with complete absence of evidence for self or environmental awareness), or minimally conscious state (MCS) (defined as a state of severely altered consciousness in which minimal but clearly discernible behavioral evidence of self- or environmental awareness is demonstrated)^48^. Finally, the NeuroPSI (a short neuropsychological test battery for use with Spanish-speaking adults)^49^ and the Montreal Cognitive Assessment (MoCA) test^50^ were performed by a former neuropsychologist (C.P.H.) for cognitive function assessment at 3- and 6-month follow-ups whenever possible according to the patient's condition or a fatal outcome. However, MoCA test was not used as an endpoint for analysis in this study. Endpoints for evaluation were defined as an early consciousness status based on the average GCS score for the previous two days before ICU discharge or a fatal outcome. The GCS was used as dichotomous for univariate analysis as the final goal was to evaluate a favorable or not favorable outcome (those with GCS score ≤ 8/15 and those with a score > 8), while for the multivariate analysis was based on the raw score as the goal was looking for the GCS score at the ICU discharge.

### Neuroimaging data acquisition

A 1.5-T General Electric scanner was used for data acquisition. As reported previously^12^, we acquired one hundred and eighty multislice T2*-weighted functional images using an axial slice orientation and covering the whole brain (slice thickness = 4.5 mm without free space, matrix = 64 × 64 mm, TR = 3000 ms, TE = 60 ms, flip angle = 90° and FOV = 288 × 288 mm). The three initial volumes were discarded to avoid T1 saturation effects. Moreover, axial DWI (slice thickness = 2.5 mm without free space, matrix = 100 × 100, TR = 17,000 ms, TE = 96 ms, flip angle = 90°, FOV = 250 × 250 mm, b value = 1000 and gradient directions = 30, voxel size = 2.4 × 2.4 × 4.5 mm) was acquired. Finally, structural axial T1 (slice thickness = 1 mm, GAP = 1 mm, matrix = 256 × 256 mm, TR = 670 ms, TE = 22 ms, flip angle = 20° and FOV = 250 × 250 mm, voxel size = 1 × 1 × 1.2 mm) and axial T2 (slice thickness = 6 mm, GAP = 1 mm, matrix = 320 × 320 mm, TR = 6.000 ms, TE = 96 ms, flip angle = 90° and FOV = 220 × 220 mm) images were acquired for anatomical reference.

### Neuroimaging data preprocessing

The T1 and rsfMRI data were preprocessed using the approach suggested by Kandeepan et al.^51^ In particular, T1 preprocessing included manual removal of the neck, brain extraction using FSL^52^, correction of low-frequency intensity nonuniformity based on the N4 bias field correction algorithm from SimpleITK^53^, image denoising based on the nonlocal means algorithm from Dipy^53,54^, and spatial normalization to standard stereotactic Montreal Neurological Institute (MNI) space using the SPM12 normalization algorithm^53–55^. The initial six volumes of the fMRI data were discarded to avoid T1 saturation effects. Head motion and slice timing corrections were performed on the fMRI data using FSL, followed by artifact correction using RapidArt^56^. Subsequently, the fMRI data were coregistered to a T1 image using SPM12 and spatially normalized to the MNI space using the SPM12 normalization algorithm. Finally, spatial smoothing of the fMRI data was performed with a Gaussian kernel of 8 mm full width at half maximum, as implemented in SPM12. The co-registration required correction for two patients only. This correction was done using the manual registration tool available from SPM12. The rest of the processing steps were satisfactorily completed for the rest of the patients. The spurious variance was reduced by regression of nuisance waveforms derived from time series extracted from regions of noninterest (WM and cerebrospinal fluid). Additional nuisance regressors included the blood oxygen level-dependent imaging (BOLD) time series averaged over the whole brain. The DWI images were preprocessed using the approach suggested by Parra-Morales et al.^12^. This process included automatic realignment, correction of eddy-current artifacts by using FSL tools, reslicing to obtain the isotropic voxel size, automatic brain extraction by the BET tool from FSL, and improvement of signal-to-noise rate using the Non-Local mean algorithm from Dipy. Over more, a DTI model was fixed to the diffusion data images based on the Dipy implementation^54^ and the standard scalar maps derived from the DTI model were computed, in particular, the fractional anisotropy (FA), mean diffusivity (MD), radial diffusivity (RD), and axial diffusivity (AD). Finally, the data imaging corresponding to the control group was used for calibration of each stage of the neuroimaging preprocessing. The output for each step proposed in the preprocessing pipeline was reviewed through visual inspection by J.R, D.M., and F.G. in order to identify inappropriate results. Manually realignment was required for specific subjects.

### Location and characterization of regions of interest

ROIs for AAN reconstruction were located based on Harvard Ascending Arousal Network Atlas provided by the Martinos Center for Biomedical Imaging, Charleston, Massachusetts, USA^14^, and the cortex nuclei were extracted from Harvard–Oxford Atlas provided by the Harvard Center for Morphometric Analysis^57^. These nuclei were linearly (rigid, translation, and affine transformation) and nonlinearly (symmetric diffeomorphic registration)^58^ registered with each subject space.

### AAN functional connectivity

Many features were extracted from the distinct imaging acquisition techniques to characterize the connectivity of the AAN with the rest of the brain. Functional connectivity (FC) was estimated using a measure of Pearson’s correlation among the average filtered time courses of eight AAN nuclei and forty-eight cortical nuclei^57^. A bandpass Butterworth filter with cutoff frequencies set at 0.005 Hz and 0.1 Hz was used for this step^59^, which produced three hundred eighty-four FC values (8 AAN nuclei^14^ × 48 cortical nuclei^57^). These sets of values were summarized by averaging the quantities to eight representative values associated with each AAN nucleus. The average functional connectivity between each AAN nucleus and the cortex was used for this purpose.

### AAN structural connectivity

A constant solid angle model was used to obtain directions from diffusion imaging^60^. This model estimates the orientation distribution function (ODF) at each voxel. This ODF is a function of the distribution of water movement, and its peaks are a suitable estimate for the orientation of each tract at each voxel. Afterwards, a deterministic local fiber tracking algorithm was applied for the WM^61^, with the following set of parameters: min separation angle = 30°, step size = 1 and number of seeds in each voxel = 8. Subsequently, the number of tracts that may connect every possible pair of nuclei between the AAN and the cortex was calculated (8 AAN nuclei^14^ × 48 cortical nuclei^57^). These sets of values were also averaged to obtain the eight representative quantities associated with each AAN nucleus.

### AAN DTI scalar values

For each AAN nuclei were computed the FA, MD, RD, and AD based on a DTI model (32 metrics). Recently, metrics based on DTI scalar maps showed changes inside gray matter in pathological conditions, in particular, these correlated with tissue compression^34^. This is a novel use of scalars values based on the DTI model which traditionally had been used just inside white matter for patients with several brain damage^62^.

### Statistical analysis

#### Univariate analysis

To assess the predictive ability of image-based features, univariate analysis of each single value from the set of 60 measurements [average functional connectivity for each AAN nuclei (8 values), average track number across each AAN nuclei (8 values); FA, MD, RD, AD for each AAN nuclei (32 values); FA, MD, RD, AD for grey matter, white matter, and whole brain (12 values)] was performed through a receiver operating characteristic (ROC) curve (Fig. 2). The measurements were grouped according to the diagnosis, and the outcome was established as indicated in the previous section. The associated area under the curve was then used to determine the predictive ability of a single image-based feature linked to the admission diagnosis.

#### Multivariate analysis

A general linear model (GLM) was employed for multivariate analysis. GLM is able to quantify the variation of a dependent variable in terms of a linear combination of several reference independent variables^63^. GLM was implemented as follows:

$${Y}_{outcome}={\beta }^{fMRI}{X}^{fMRI}+{\beta }^{DTI}{X}^{DTI}+{\beta }^{confoundings}{X}^{confoundings}+e$$

where ${Y}_{outcome}$ is a column vector that contains the mean GCS score for the last two days before ICU discharge; ${X}^{fMRI}$, ${X}^{DTI}$ and ${X}^{confoundings}$ are matrices that contain for each column the quantitative value for each feature from fMRI, DTI and confounding data (patient age, etiology, ICU length of stay, ICU admission, presence of infection during ICU stay, time elapsed since CPA, the need for decompressive craniectomy, use of anticonvulsants, and days under inotropic/vasopressor support), respectively; $e$ represents the error during model fit; ${\beta }^{fMRI}$, ${\beta }^{DTI}$ and ${\beta }^{confoundings}$ represent the weight in the registration model for each fMRI, DTI and confounding variable. The R^2^ value was used to determine the level of model fit for each case. Finally, *p*-values were used to determine the significance level for each variable explaining dependent variables. In order to control multiple comparisons, p values were adjusted according to the number of values compared in the analysis^64^. Endpoints for multivariate analysis were the same used for the univariate analysis.

## Supplementary information

Supplementary Information.

## Data Availability

The data that support the findings of this study are available on request from the corresponding author, [JHM]. The data are not publicly available due to the containing information that could compromise the privacy of research participants (e.g. Patients' names, surrogates' names, IDs).
