# Supplementary material for: Structural and functional connectivity of the ascending arousal network for prediction of outcome in patients with acute disorders of consciousness
Source: Sci Rep. 2021 Nov 25;11:22952. doi: 10.1038/s41598-021-98506-7 (PMC8617304; doi:10.1038/s41598-021-98506-7)
Supplement: Supplementary file 1 — Supplementary Information. [file 41598_2021_98506_MOESM1_ESM.docx]

**Supplements/Appendices**

**Appendix 1. Measurements of functional network connectivity in patients with disorders of consciousness after traumatic brain injury.**

| **Measurement** | **WM** | **GM** | **WB** | **DR** | **LC** | **MRF** | **MR** | **PGM** | **PC** | **PON** | **VTA** |
| --- | --- | --- | --- | --- | --- | --- | --- | --- | --- | --- | --- |
| Fractional Anisotropy | 0.520 | **0.800** | 0.720 | 0.360 | 0.400 | 0.240 | 0.400 | 0.320 | 0.400 | 0.400 | 0.200 |
| Mean Diffusivity | 0.360 | 0.360 | 0.560 | 0.480 | 0.520 | 0.640 | 0.520 | 0.560 | 0.520 | 0.520 | 0.760 |
| Axial Diffusivity | 0.400 | 0.360 | 0.520 | 0.480 | 0.480 | 0.320 | 0.600 | 0.520 | 0.440 | 0.600 | 0.640 |
| Radial Diffusivity | 0.360 | 0.400 | 0.560 | 0.520 | 0.640 | 0.680 | 0.560 | 0.640 | 0.480 | 0.640 | **0.800** |
| Functional Network Connectivity | NA | NA | NA | 0.120 | 0.400 | 0.640 | 0.400 | 0.520 | 0.240 | 0.400 | 0.200 |
| Number of fibers | NA | NA | NA | 0.760 | 0.560 | 0.360 | 0.440 | 0.600 | 0.160 | **0.880** | 0.440 |

WM = white matter, GM = gray matter, WB = whole brain, DR = dorsal raphè, LC = locus coeruleus, MRF = midbrain reticular formation, MR = median raphè, PGM = periaqueductal gray matter, PC = parabranchial complex, PON = pontis oralis nucleus, VTA = ventral tegmental area

**Appendix 2. Measurements of functional network connectivity in patients with disorders of consciousness after cardiac arrest treated within our institution with successful resuscitation with hypoxic brain ischemia.**

| **Measurement** | **WM** | **GM** | **WB** | **DR** | **LC** | **MRF** | **MR** | **PGM** | **PC** | **PON** | **VTA** |
| --- | --- | --- | --- | --- | --- | --- | --- | --- | --- | --- | --- |
| Fractional Anisotropy | **0.800** | 0.550 | 0.400 | 0.750 | 0.600 | 0.650 | 0.450 | 0.750 | 0.400 | 0.400 | **0.900** |
| Mean Diffusivity | **0.850** | 0.750 | 0.750 | 0.200 | 0.250 | 0.350 | 0.500 | 0.350 | 0.450 | 0.675 | 0.350 |
| Axial Diffusivity | **0.850** | 0.700 | 0.750 | 0.350 | 0.350 | 0.525 | 0.650 | 0.550 | 0.400 | 0.350 | 0.500 |
| Radial Diffusivity | **0.850** | **0.850** | 0.750 | 0.250 | 0.400 | 0.300 | 0.550 | 0.350 | 0.400 | 0.500 | 0.100 |
| Functional Network Connectivity | NA | NA | NA | 0.250 | 0.550 | 0.700 | 0.450 | 0.500 | 0.600 | 0.650 | 0.350 |
| Number of fibers | NA | NA | NA | 0.600 | 0.700 | 0.450 | 0.725 | 0.600 | 0.650 | 0.400 | 0.350 |

WM = white matter, GM = gray matter, WB = whole brain, DR = dorsal raphè, LC = locus coeruleus, MRF = midbrain reticular formation, MR = median raphè, PGM = periaqueductal gray matter, PC = parabranchial complex, PON = pontis oralis nucleus, VTA = ventral tegmental area

**Appendix 3. Measurements of functional network connectivity in patients with disorders of consciousness after stroke.**

| **Measurement** | **WM** | **GM** | **WB** | **DR** | **LC** | **MRF** | **MR** | **PGM** | **PC** | **PON** | **VTA** |
| --- | --- | --- | --- | --- | --- | --- | --- | --- | --- | --- | --- |
| Fractional Anisotropy | 0.583 | 0.417 | 0.417 | 0.562 | 0.458 | 0.417 | 0.292 | 0.583 | 0.625 | 0.292 | 0.750 |
| Mean Diffusivity | 0.458 | 0.531 | 0.417 | 0.396 | 0.396 | 0.458 | 0.667 | 0.271 | 0.375 | 0.771 | 0.354 |
| Axial Diffusivity | 0.458 | 0.542 | 0.396 | 0.438 | 0.375 | 0.562 | 0.438 | 0.312 | 0.437 | 0.490 | 0.646 |
| Radial Diffusivity | 0.521 | 0.500 | 0.417 | 0.396 | 0.333 | 0.458 | 0.729 | 0.333 | 0.333 | 0.729 | 0.302 |
| Functional Network Connectivity | NA | NA | NA | 0.646 | 0.667 | 0.271 | 0.687 | 0.479 | 0.750 | 0.729 | **0.875** |
| Number of fibers | NA | NA | NA | 0.479 | 0.677 | 0.479 | 0.396 | 0.750 | 0.438 | 0.271 | 0.729 |

WM = white matter, GM = gray matter, WB = whole brain, DR = dorsal raphè, LC = locus coeruleus, MRF = midbrain reticular formation, MR = median raphè, PGM = periaqueductal gray matter, PC = parabranchial complex, PON = pontis oralis nucleus, VTA = ventral tegmental area

**Appendix 4. Variables and descriptive analysis**

***Variables***

*DTI-Based Variables*

A set of DTI scalar values was measured for each AAN nucleus. Features of the fractional anisotropy (FA), axial diffusivity (AD), radial diffusivity (RD) and mean diffusivity (MD) of the WM, GM, and whole brain were measured. In addition, the number of fibers, structural connectivity and functional network connectivity for each AAN nucleus were computed for each patient, resulting in a set of 60 imaging-based features. Each single feature was used to evaluate prediction capability; however, the features were grouped by region, i.e., the WM, gray matter, whole brain and eight thalamus nuclei, to perform adequate comparison. An outcome value was assigned given the endpoints mentioned above. We applied a generalized linear model that considered all variables, including those with the most predictive value as well as covariates aiming to predict consciousness outcome based on imaging data.

*Confounding Variables*

The following variables were considered to be possible confounders for the predictive model: (1) patient age, (2) etiology, (3) ICU length of stay (LOS), (4) ICU admission, (5) presence of infection during ICU stay, (6) time elapsed since CPA (if present), (7) the need for decompressive craniectomy, (8) use of anticonvulsants, and (9) days under inotropic/vasopressor support (when applicable).

*Descriptive Analysis*

A sample size of 50 patients was calculated, which included an additional 20% for possible losses. A proportion of findings in 50% of the images of patients who may not recover consciousness and 10% of the images in those who may recover consciousness were assumed for the analysis. The following parameters were used: alpha level = 5% and power = 80%. Student's t test, the Mann-Whitney test, the chi-square test, and the exact test were used accordingly. For functional connectivity, tools were developed for the software and used for this purpose. Correction for multiple comparisons was performed when required by using the Bonferroni correction approach.
